# Determining breast cancer histological grade from RNA-sequencing data

**DOI:** 10.1186/s13058-016-0710-8

**Published:** 2016-05-10

**Authors:** Mei Wang, Daniel Klevebring, Johan Lindberg, Kamila Czene, Henrik Grönberg, Mattias Rantalainen

**Affiliations:** Department of Medical Epidemiology and Biostatistics, Karolinska Institutet, Nobels Vag 12A, Stockholm, 171 77 Sweden

**Keywords:** Breast neoplasms, Nottingham histologic grades, RNA-seq, Isoform

## Abstract

**Background:**

The histologic grade (HG) of breast cancer is an established prognostic factor. The grade is usually reported on a scale ranging from 1 to 3, where grade 3 tumours are the most aggressive. However, grade 2 is associated with an intermediate risk of recurrence, and carries limited information for clinical decision-making. Patients classified as grade 2 are at risk of both under- and over-treatment.

**Methods:**

RNA-sequencing analysis was conducted in a cohort of 275 women diagnosed with invasive breast cancer. Multivariate prediction models were developed to classify tumours into high and low transcriptomic grade (TG) based on gene- and isoform-level expression data from RNA-sequencing. HG2 tumours were reclassified according to the prediction model and a recurrence-free survival analysis was performed by the multivariate Cox proportional hazards regression model to assess to what extent the TG model could be used to stratify patients. The prediction model was validated in *N*=487 breast cancer cases from the The Cancer Genome Atlas (TCGA) data set. Differentially expressed genes and isoforms associated with HGs were analysed using linear models.

**Results:**

The classification of grade 1 and grade 3 tumours based on RNA-sequencing data achieved high accuracy (area under the receiver operating characteristic curve = 0.97). The association between recurrence-free survival rate and HGs was confirmed in the study population (hazard ratio of grade 3 versus 1 was 2.62 with 95 % confidence interval = 1.04–6.61). The TG model enabled us to reclassify grade 2 tumours as high TG and low TG gene or isoform grade. The risk of recurrence in the high TG group of grade 2 tumours was higher than in low TG group (hazard ratio = 2.43, 95 % confidence interval = 1.13–5.20). We found 8200 genes and 13,809 isoforms that were differentially expressed between HG1 and HG3 breast cancer tumours.

**Conclusions:**

Gene- and isoform-level expression data from RNA-sequencing could be utilised to differentiate HG1 and HG3 tumours with high accuracy. We identified a large number of novel genes and isoforms associated with HG. Grade 2 tumours could be reclassified as high and low TG, which has the potential to reduce over- and under-treatment if implemented clinically.

**Electronic supplementary material:**

The online version of this article (doi:10.1186/s13058-016-0710-8) contains supplementary material, which is available to authorized users.

## Background

Histologic grade (HG) is considered as one of the best established prognostic factors in breast cancer diagnostics [1]. According to the Nottingham grading system, breast cancer is categorised to three HGs depending on the degree of tumour cell differentiation: well differentiated (grade 1), moderately differentiated (grade 2) and poorly differentiated (grade 3) [2, 3]. The grading system assesses three dimensions: tubule formation (tubularity), nuclear pleomorphism (nuclearity) and mitotic count. Each component is categorised as a score from 1 to 3. The overall grade is determined by the sum of the scores from the three components. Tumours with a higher grade are associated with a lower survival rate [4].

A morphological assessment of biological characteristics provides important information related to the clinical behaviour of breast cancer. Patients with grade 3 tumours are recommended for adjuvant chemotherapy, whereas patients with grade 1 tumours are often oestrogen receptor (ER) positive, and thus amenable for a less toxic endocrine therapy [1]. In general, half of the cases are assigned to grade 1 or 3. Grade 2 tumours account for the other half and they are associated with an intermediate risk of recurrence, which is not informative for clinical decision-making [5]. Furthermore, inter-pathologist variability in the morphological assessment contributes to a degree of uncertainty in tumour grade classification [6, 7].

In the last decade, genome-wide gene expression profiling methods have introduced new ways for tumour classification using molecular signatures. The genomic grade index (GGI) was previously proposed as a method to stratify ER-positive grade 2 tumours into two groups [5], which could be potentially integrated to the current HG system. GGI is based on a 97-gene signature with gene expression abundances quantified by microarray technology. A large cohort study revealed that GGI provides significant prognostic information beyond clinical characteristics including tumour size, lymph node status and HG [8] in ER-positive tumours. The study indicated that combining molecular signatures with HG may improve the prognostic power.

The 97-gene biomarker panel used for GGI was developed based on microarray data at the gene level. Recently next-generation sequencing of RNA (RNA-seq) has emerged as the de facto standard for gene-expression profiling, also enabling quantification of gene expression at both gene and isoform level. Isoform-level gene expression data has the potential to provide further insight and prognostic information beyond gene-level expression data. For example, it has been found that different isoforms may have different molecular functions [9]. In prostate cancer, it has been reported that two isoforms of *KLF6* lead to increased cell growth and an increased risk of prostate cancer [10].

To improve patient stratification and to enable better personalised care for breast cancer patients, we developed methods based on RNA-seq data to determine the transcriptomic grade (TG) of tumours. The TG model we propose dichotomises tumours into a high grade and low grade, thus providing improved stratification of the intermediate HG2 patients. The proposed method has the potential to reduce both over- and under-treatment of HG2 patients. We also characterise the molecular basis of HG by investigating to what extent RNA-seq gene- and isoform-level expression are associated with HG.

## Methods

### Data sets and subjects

#### Clinseq

Study participants were 275 females diagnosed with primary invasive breast cancer from the Clinseq study (Clinical Sequencing of Cancer in Sweden; http://clinseq.org/) [11]. The Clinseq breast cancer study comprises two Swedish cohorts, Libro-1 [12] and Karma [13]. Study participants from Karma were recruited perspectively from 2012 in Stockholm South General Hospital (in Swedish: Södersjukhuset). Study participants from Libro-1 were recruited retrospectively among patients who underwent surgery between 2001 and 2008 at the Karolinska University Hospital (in Swedish: Karolinska Universitetssjukhuset) and were alive in 2009. The study is approved by the Ethical Committee of the Karolinska Institute (reference number 2013/1833-31/2) and all participants provided written informed consent.

Primary tumour tissues were collected from the participants and stored in the Karolinska Institute Biobank. The HGs of cancer were evaluated by pathologists based on the Nottingham grading system [2, 3]. Grade information was extracted from the patient pathology records. Clinical and follow-up information was retrieved through a link to the Swedish national breast cancer register, the Information Network for Cancer Care [14] and the regional cancer centres [15]. Clinical biomarkers – ER, progesterone receptor (PR), human epidermal growth factor receptor 2 (HER2) and KI67 – were measured by an immunohistochemistry assay. ER and PR status were determined as positive if comprising more than 10 % of the corresponding nuclear staining. The cut-off for KI67 was 20 % positively stained tumour cells. HER2 status was classified as positive if a fluorescent in situ hybridisation (FISH) result indicated amplification or, in the absence of a FISH result, if the sample was graded 3+ by the immunohistochemistry assay.

#### The Cancer Genome Atlas

We also used RNA-seq data from The Cancer Genome Atlas (TCGA) (http://cancergenome.nih.gov/). Unaligned RNA-seq data (FASTQ format) of 1126 invasive breast carcinoma samples were downloaded in June 2014 after approval from the TCGA data access committee (dbGAP project ID 5621). The grade information was manually extracted from copies of patient pathology reports provided by TCGA. The HGs of TCGA breast cancer patients were diagnosed with multiple grading systems. To ensure the consistency with our study population, only 487 female breast cancer patients from TCGA whose HG was diagnosed by the Nottingham grading system and for which all three subcomponent scores were available were included in this study. We acknowledge that because scoring was by multiple pathologists across multiple institutions, that there may still be some variation in grades. Bioinformatic preprocessing of the RNA-seq data used identical methods as for the CLINSEQ data set (described below).

### RNA-sequencing

RNA from breast tissue was extracted from fresh frozen breast tumour tissues that were removed during surgery. RNA was extracted using an AllPrep DNA/RNA/Protein mini kit (Qiagen, Germany). RNA was assessed using Bioanalyzer (Agilent, US) to ensure high quality (RNA integrity number >8). Then, 1 µg of total RNA was used for rRNA depletion using RiboZero (Illumina, US) and stranded RNA-seq libraries were constructed using a TruSeq Stranded Total RNA Library Prep Kit (Illumina, US). Then 2×100 paired-end sequencing was performed on an Illumina HiSeq 2500 (Illumina, US) at the Science for Life Laboratory (Stockholm, Sweden). The insert sized ranged from approximately 50 to 300 bp. The resulting RNA-seq reads were aligned to the reference genome (GRCh37.73) using STAR [16] version-2.4.0e39 with the following parameters: -outSAMmapqUnique 50, to set the maximum alignment quality score to 50; -outSAMunmapped Within, to include unmapped reads in the resulting SAM file; -chimSegmentMin 20 to require that a minimum of 20 bases map to each end of a chimeric transcript (output in a separate file) and -outSAMattributes NH HI AS nM NM MD XS to include additional attributes in the SAM file. Gene-level expression was quantified using HTSeq-count [17] version-0.6.040 with the following parameters: -stranded = no and -mode = intersection-nonempty for counting reads using the default alignment quality filter threshold of 10.

There are 20,477 genes in the reference genome and 144,027 isoforms in the reference transcriptome. There were 18,795 genes with non-zero read counts. Isoform-level expression was quantified using Sailfish version 0.6.3 [18] and ENSEMBL version 75 with the following parameters: -p 16 -k 20 to use 16 threads and a *k*-mer size of 20. For each sample, Sailfish version 0.6.3 was run with default parameters except for library type, which was set to --libtype”T=PE:S=AS:O=><” for paired-end second-read mapping to the antisense strand and inwards orientation. The default bias correction was applied. Isoforms were filtered if they failed to achieve counts per million of 1 in 75 % of the samples. After filtering, there were 42,718 isoforms left for downstream analysis.

RNA-seq read counts were scaled logarithmically by the variance stabilising transformation implemented in DESeq2 for prediction modelling [19]. For differential expression (DE) analysis, read counts were normalised by the TTM method implemented in R package edgeR [20, 21].

### Prediction models

#### Transcriptomic grade

We applied the multivariate elastic-net penalised logistic regression model [22] for prediction of tumour grade with either transcriptome-wide gene- or isoform-level normalised expression values as predictors. The elastic-net method is implemented in the R package glmnet [23]. The tumour grade model was trained on HG1 and HG3 tumours and we estimated separate models for gene- and isoform-level transcriptomic data. The two models are referred to as TG at gene level (TG-Gene) and at isoform level (TG-Iso).

A nested cross-validation (CV) procedure was used to estimate prediction performance while also optimising model parameters (alpha and lambda). For outer CV, class-balanced Monte-Carlo CV was performed (100 rounds). The training/test set ratio was 90 %/10 %. Patients with grade 3 or grade 1 were balance stratified into the training and test sets. The CV samples were identical across the evaluation of different models to ensure accurate model comparison. The parameters were optimised empirically based on the outer CV loop training set in the inner CV (10 × tenfold CV). The alpha parameter was evaluated on a grid at the following points: 0.001, 0.005, 0.01, 0.05, 0.1, 0.2, 0.3, 0.5, 0.7 and 0.9. The best alpha and lambda were chosen based on minimising the average misclassification error. The probability of being HG3 was calculated for outer CV test set observations from the optimised model in each CV round.

Receiver operating characteristic (ROC) curves of TG against true HGs were constructed. The area under the ROC curve (AUC) and 95 % confidence interval (CI) were generated to compare model performance. The AUCs of ROC curves were compared by the DeLong test [24]. The decision boundary was determined at the point closest to the top-left part of the ROC curve using the pROC package for R [25]. HG2 patients were classified as high risk (HG2-High) if their predicted probability was larger than or equal to the cut-off point; otherwise, patients were classified as HG2-Low.

#### TG based on subcomponents of HG

We also developed prediction models based on separate modelling of the subcomponents of HG at gene- and isoform-level (SC-Gene and SC-Iso). The procedures for parameter optimisation and CV were the same as for the TG-Gene and TG-Iso models; however, they were conducted for each component separately. The three subcomponents of HG (tubularity *T*, nuclearity *N* and mitotic count *M*) were predicted by an elastic-net penalised linear regression model. For each outer CV, 90 % of the sample were selected in the training set. The proportions of HG1 and HG3 were kept as in the whole sample set. The linear multivariate model was built guided by sub-scores 1, 2 and 3. The final score was defined as the sum of the predicted subcomponent scores.

#### Genomic Grade Index

For comparison, we also implemented the previously reported GGI method [5]. According to Sotiriou’s study, the top 128 DE probes of 97 unique genes were selected to calculate GGI, and out of these we could match 96 gene symbols to genes in our data set. We applied the GGI algorithm as described in the original article [5]. We applied an identical Monte Carlo CV procedure for the GGI model as described in the previous section. Standardisation parameters (scale and offset) were generated for each training set. Then the GGI of samples in the test set were standardised using parameters from the training set, ranging from −1 to 1. AUC and 95 % CI were calculated using the ROC curve on the GGI against the true HG.

#### Validation in the secondary data set

The prediction models (TG-Gene, TG-Iso, SC-Gene and SC-Iso) were validated with the TCGA breast cancer data set [26]. The prediction models were estimated based on the Clinseq data set where model parameters (alpha and lambda) were optimised by tenfold CV for 100 times. The parameters alpha and lambda were chosen so that the mean of deviance residuals was minimised. To reduce any potential batch differences between them, the data sets were mean-centred before analysis. According to the GGI method, the index was standardised within each data set. Hence, cross data set validation does not apply to the GGI method.

### Survival analysis on HG and TG

The recurrence-free survival (RFS) rate was compared among patients with different HGs to investigate whether grade is an indicator of prognosis in this study population. The predicted high TG and low TG groups within grade 2 tumours (HG2-High and HG2-Low) were also compared.

A recurrence event is considered to be a local or regional tumour relapse, distant metastasis, contralateral tumour or death by any cause. Patients who died before experiencing a tumour metastasis were assumed to have had undetected metastasis before death [27]. The time to event is measured from the diagnostic date to the date of the first documented local or regional relapse, distant metastasis, contralateral tumour, death or last follow-up.

A Kaplan–Meier curve was used to estimate the survival outcomes and groups were compared with the non-parametric log-rank statistic. Data from the two data sets (Clinseq and TCGA) were pooled together. Univariate and multivariate Cox proportional hazards regression models were fitted at time-on scale. Unadjusted and adjusted hazard ratios (HRs) and 95 % CI were calculated. In the multivariate Cox regression model, we adjusted for age, tumour size, lymph node status and ER status, and stratified by data set. Age was treated as a continuous variable. Tumour size was dichotomised based on the diameter of the tumour as ≥20 mm or <20 mm. Lymph node status was dichotomised as with or without lymph node metastases. Proportional hazards assumptions were confirmed using Schoenfeld residuals. The survival analysis was conducted using standard functions implemented in R [28, 29].

### DE analysis on HGs and subcomponents of grades

RNA-seq data were compared among patients with different HGs and subcomponents of grades to determine DE genes and isoforms. Read counts were transformed to log-counts with a precision weight by estimating the mean-variance relationship (voom) [30]. Empirical Bayes moderated *t*-statistics was applied to analyse DE isoforms. The Benjamini and Hochberg false discovery rate (FDR) was used to adjust for multiple testing [31]. The DE of genes or isoforms was defined as those with FDR-adjusted *p*<0.05. The DE analysis was performed by functions in the R package limma [32].

### Pathway analysis

A pathway enrichment analysis of DE genes based on the Reactome database (http://www.reactome.org/) [33] was conducted with R package ReactomePA [34]. Pathway overrepresentation was tested by a hypergeometric model [35].

### PAM50 subtyping

PAM50 intrinsic subtypes [36] were assigned using the nearest shrunken centroid classifier [37] in the Clinseq data set. The R package pamr was utilised to train the classifier. Optimisation (amount of shrinkage) was determined by tenfold CV selecting the parameter value based on the minimal classification error. The subtypes in TCGA were referred to their original breast cancer publication [26]. The normal-like subtype was not included as the clinical relevance for this subtype has been questioned [38]. The distributions of subtypes between HGs and predicted groups were compared by a chi-squares test or a Fisher exact test if the expected value in any cell was smaller than 5.

## Results

A description of the clinical characteristics and HG of subjects in both the Clinseq and TCGA data sets is listed in Table 1. The distributions of HGs in the two data sets were similar (*p*>0.05, chi-squares test). The mean of the patients’ ages was not statistically different across the two data sets (*p*>0.05, Student’s *t*-test). There were more tumours with a larger size and positive lymph node in the TCGA data set (*p*<0.05, chi-squares test). The distributions of ER, PR and HER2 between the two data sets were not different.

**Table 1:** Clinical characteristics of subjects in the Clinseq and TCGA data sets

	Clinseq	TCGA
*N*	275	487
Median age (range)	61 (28–94)	57 (26–90)
Histologic grade		
Grade 1 (%)	39 (14.2 %)	64 (13.1 %)
Grade 2 (%)	121 (44.0 %)	228 (46.8 %)
Grade 3 (%)	115 (41.8 %)	195 (40.0 %)
Tumour size		
≥20 mm (%)	150 (54.6 %)	317 (65.1 %)
<20 mm (%)	125 (45.5 %)	170 (34.9 %)
Lymph node status		
Positive (%)	39 (14.2 %)	260 (53.4 %)
Negative (%)	136 (85.8 %)	227 (46.6 %)
ER status		
Positive (%)	231 (84.0 %)	384 (78.9 %)
Negative (%)	42 (15.3 %)	102 (20.9 %)
NA (%)	2 (0.7 %)	1 (0.2 %)
PR status		
Positive (%)	175 (63.6 %)	334 (68.6 %)
Negative (%)	98 (35.6 %)	151 (31.0 %)
NA (%)	2 (0.7 %)	2 (0.4 %)
HER2 status		
Positive (%)	44 (16.0 %)	60 (12.3 %)
Negative (%)	225(81.8 %)	274 (56.3 %)
Equivocal (%)	–	108 (22.2 %)
NA (%)	6 (2.2 %)	45 (9.2 %)
KI67 status ^*a*^		
Positive (%)	131 (47.6 %)	–
Negative (%)	121 (44.0 %)	–
NA (%)	23 (8.4 %)	–

*ER* oestrogen receptor, *NA* not applicable, *PR* progesterone receptor, *TCGA* The Cancer Genome Atlas

^a^Measurement of KI67 is not available for the TCGA data set

### HG can be predicted from RNA-seq gene expression profiles

**Fig. 1:**
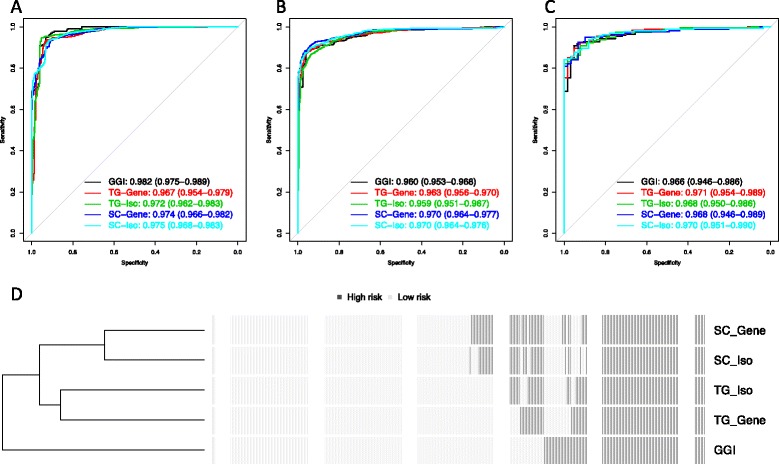
Prediction models comparison. ROC curves of CV on five models: GGI, TG-Gene, TG-Iso, SC-Gene and SC-Iso for **a** the Clinseq data set and **b** the TCGA data set. AUC estimates and 95 % CI of ROC curves are listed for each model. **c** Cross-data set validation of multivariate prediction models (TG-Gene, TG-Iso, SC-Gene and SC-Iso). Models were estimated based on the Clinseq data set, and grade in the TCGA data set was predicted. **d** Predictions of HG2 tumours by five models for all observations in the Clinseq and TCGA data sets. *AUC* area under the ROC curve, *CI* confidence interval, *CV* cross-validation, *GGI* genomic grade index, *ROC* receiver operating characteristic, *TCGA* The Cancer Genome Atlas

We developed prediction models based on HG3 and HG1 individuals, using RNA-seq data at both gene and isoform level (TG-Gene, TG-Iso, SC-Gene and SC-Iso). The numbers of predictors selected in each final model (TG-Gene in Clinseq, TG-Gene in TCGA, TG-Iso in Clinseq and TG-Iso in TCGA) were 427, 96, 112 and 255, respectively listed in Additional file 2. The GGI method was also implemented for comparison. Prediction performance, as assessed by ROC curves, was found to be similar for all five models within each data set (Fig. 1a, b). In the Clinseq data set, the AUC of the GGI method was higher than for the SC-Iso model (*p*<0.05, DeLong test). The AUC of the ROC curve for the SC-Gene, SC-Iso, TG-Gene and TG-Iso models showed no statistical difference (*p*>0.05, DeLong test). For the TCGA data set, the AUCs of the SC-Gene and the SC-Iso models were higher than for any of the TG-Gene, GGI and TG-Iso models (*p*<0.05, DeLong test).

Next, we assessed the concordance of the five different models in the classification of HG1 and HG3 by predicting all observations by the fitted models. The results indicated a relatively high degree of concordance across all methods (Additional file 1: Figure S1).

To validate the prediction models further, they were estimated based on the Clinseq data set, and the grade in the TCGA data set was predicted. The ROC curves of the models are in Fig. 1c. All of the models achieved high accuracy (AUC = 0.97), and vice versa, when models were trained in TCGA and predicted in Clinseq (Additional file 1: Figure S8).

We then investigated if patients with a grade 2 tumour were classified consistently into high and low TG groups by the prediction models. The concordance of the models is displayed in Fig. 1d. Among the five models, 76.4 % (252 of 330 individuals) of the HG2 patients were classified consistently (HG2 patients from both the Clinseq and TCGA data sets). Given that patients clinically classified as HG2 are considered as intermediate in current clinical practice, with little or no impact on clinical decision-making due to their intermediate status, 76 % consistency across multiple different models is to be considered as a relatively high degree of concordance.

### Modelling of HG subcomponents

To investigate if the different subcomponents of histological grade were different on a gene expression level, we developed prediction models for subcomponents of grade (see ‘Methods’) and evaluated the prediction performance. The distribution of subcomponent scores of HG are summarised in Table 2. The ROC curves of three components from the SC-Gene and SC-Iso models for the Clinseq and TCGA data sets are illustrated in Additional file 1: Figure S4. We found that the molecular information in the RNA-seq data (gene or isoform level) enabled a good ability to classify score 1 and score 3 individuals in terms of mitotic count (AUC = 0.92, SC-Gene model for the Clinseq data set), while the classification of score 1 and score 3 for the tubularity and nuclearity components was substantially lower (AUC = 0.68 and AUC = 0.76, SC-Gene model for the Clinseq data set), suggesting that the molecular difference between score 1 and score 3 individuals for these components is limited.

**Table 2:** Summary of HG and subcomponents of subjects

	Grade	Tubularity	Nuclearity	Mitotic counts
Clinseq				
1	39	18	4	119
2	121	55	136	73
3	115	198	131	79
Missing	0	4	4	4
TCGA				
1	64	12	24	197
2	228	91	224	119
3	195	337	195	123
Missing	0	47	44	48

*HG* histologic grade, *TCGA* The Cancer Genome Atlas

### RFS is different among HGs and TGs

**Fig. 2:**
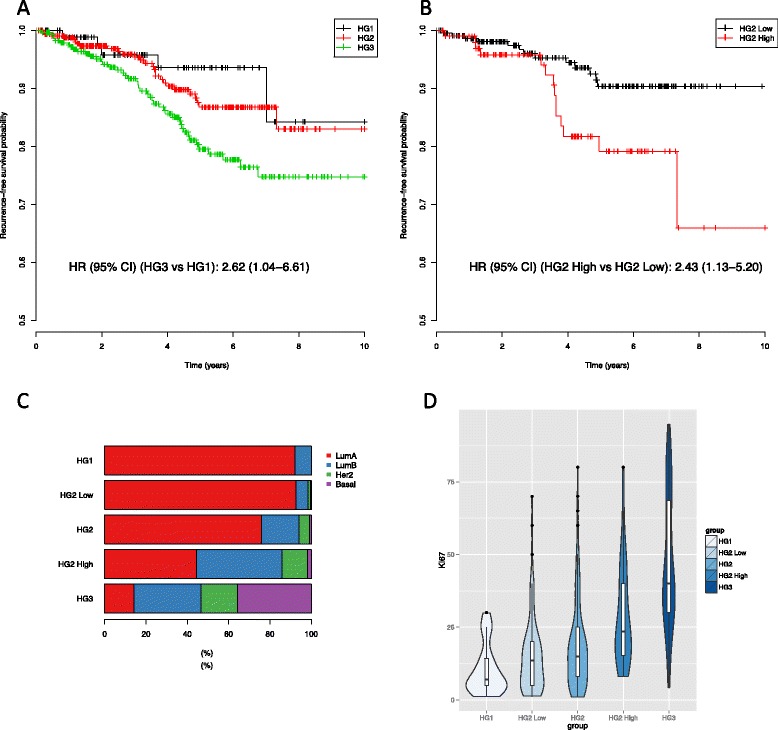
TG-Gene model predictions in HG2 tumours (Clinseq and TCGA data sets combined). **a** Kaplan–Meir curves of RFS by HGs. **b** Kaplan–Meir curves of RFS between groups predicted by the TG-Gene model (HG2-High and HG2-Low). **c** PAM50 subtype distribution of HGs and predicted groups in HG2. **d** KI67 distribution. *HG* histologic grade, *RFS* recurrence-free survival

To evaluate if the RFS rate was associated with HGs, we compared RFS between HG groups (Fig. 2a). The survival analysis was carried out on the Clinseq and TCGA data sets combined. Forest plots from the univariate and multivariate Cox regression models for each data set are displayed in Additional file 1: Figures S10 and S11. No obvious bias was found between the two cohorts. The median follow-up time was 3.6 years. The RFS rate was found to be different between HG groups (*p*=0.017, log-rank test). In the Cox regression model, the unadjusted HR of grade 3 against 1 was 2.62 (95 % CI = 1.04–6.61). The adjusted HR comparing grade 3 with grade 1 was not statistically significant (Table 3).

**Table 3:** *p* value of log-rank test and HRs of Cox regression on RFS comparing breast cancer patients with different HGs and predicted groups in HG2 tumours

	*N*	Events	Log-rank test	HR unadjusted ^a^	HR adjusted ^b^
			(*p* value)	(95 % CI)	(95 % CI)
Histologic grades					
HG1	98	6	0.017 ^∗^	1.00 (Reference)	1.00 (Reference)
HG3	294	44		2.62 (1.04–6.61) ^∗^	2.02 (0.76–5.40)
GGI					
Low risk	223	12	0.010 ^∗^	1.00 (Reference)	1.00 (Reference)
High risk	110	15		2.64 (1.23–5.70) ^∗^	3.04 (1.35–6.81) ^∗^
TG-Gene					
Low risk	228	13	0.018 ^∗^	1.00 (Reference)	1.00 (Reference)
High risk	105	14		2.43 (1.13–5.20) ^∗^	2.50 (1.14–5.50) ^∗^
TG-Iso					
Low risk	216	11	0.014 ^∗^	1.00 (Reference)	1.00 (Reference)
High risk	117	16		2.53 (1.17–5.47) ^∗^	2.64 (1.20–5.79) ^∗^
SC-Gene					
Low risk	198	14	0.584	1.00 (Reference)	1.00 (Reference)
High risk	117	11		1.25 (0.56–2.78)	1.23 (0.54–2.77)
SC-Iso					
Low risk	208	14	0.393	1.00 (Reference)	1.00 (Reference)
High risk	107	11		1.41 (0.64–3.14)	1.45 (0.65–3.24)

*GGI* genomic grade index, s*HR* hazard ratio, *RFS* recurrence-free survival

^a^HR unadjusted, only stratified by data set

^b^HR adjusted for age, tumour size, lymph node status and ER status, stratified by data set

^∗^ *p*<0.05

Next, we compared RFS rates between TGs of HG2 patients to determine if there were any evidence that the TG models provided prognostic information. RFS curves of HG2-High and HG2-Low groups for all five TG models were compared (Additional file 1: Figure S2). Groups predicted by the GGI, TG-Gene and TG-Iso models indicated statistically significant differences in RFS rate (Table 3, *p*<0.05, log-rank test).

Figure 2b shows the corresponding Kaplan–Meir curves of HG2-High and HG2-Low predicted by the TG-Gene model. The unadjusted HR of HG2-High versus HG2-Low was 2.43 (95 % CI = 1.13–5.20). When adjusted for age, tumour size, lymph node status and ER status, HR increased to 2.50 (95 % CI = 1.14–5.50).

### Association between HGs and PAM50 subtypes

We then investigated the association between HGs and the PAM50 intrinsic gene signature to determine if the subtype distribution was similar when stratified by HG and TG, focusing on reclassified HG2 tumours. Subtype proportions for patients stratified by grade predicted by the TG-Gene model are displayed in Fig. 2c. The other prediction models provided highly similar results (see Additional file 1: Figure S2). Samples from the Clinseq and TCGA data sets were combined. The distributions of subtypes between HGs were different (chi-squares = 323.3, *p*<0.001). We found the distribution of subtypes in the HG2-Low group were similar to HG1 (*p*>0.05, Fisher’s exact test). However, the subtype distributions for HG2-High were found to be different to HG3 (chi-squares = 67.3, *p*<0.001).

The distribution of PAM50 subtypes in the TG-High and TG-Low groups of all the samples was compared to HG3 and HG1, respectively. In the TG-Gene model, the distribution of subtypes in TG-Low was similar to HG1 (*p*>0.05, Fisher’s exact test). The subtype distribution in TG-High was found to be different to HG3 (*p*=0.02, chi-squares test). See Additional file 1: Figure S3 for the subtype distribution across all five prediction models.

Within subtype luminal A, the RFS rates of TG-High and TG-Low stratified by the TG-Iso model were different (*p*=0.028, log-rank test, Additional file 1: Figure S9). Suffering from the limited number of recurrent events observed, the results were not consistent among different models. A survival analysis could not be conducted in other subtypes due to the limited sample size and limited number of events.

### TG-High patients had higher proliferation levels

We further analysed the relationship between KI67, a proliferation marker, and HGs and TGs (Fig. 2d). The KI67 level was associated with HGs (*p*<0.001, ANOVA test). Comparing the KI67 of predicted groups within HG2, the mean of HG2-High is higher than HG2-Low (*p*<0.001, *t*-test). Comparing the KI67 level between the TG-High and TG-Low groups predicted from all of the patients, the mean of KI67 was higher in TG-High than TG-Low (*p*<0.001, *t*-test). The results were consistent among the five models (Additional file 1: Figures S2 and S3).

### DE genes and isoforms among HGs are associated with cell cycle

**Fig. 3:**
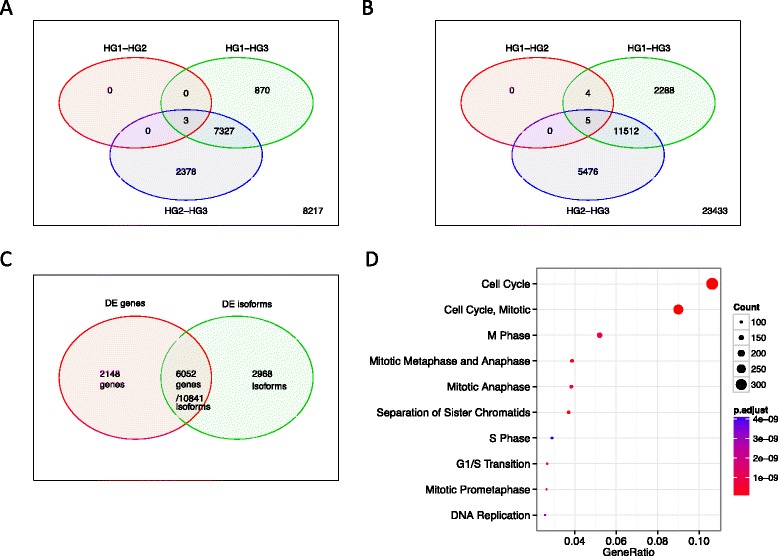
DE analysis. **a** Venn diagram of DE genes between HGs. **b** Venn diagram of DE isoforms. **c** Overlaps of DE genes and isoforms between HG1 and HG3. **d** Top ten enriched pathways of DE genes. Count is the number of genes found in each pathway. *DE* differential expression or differentially expressed, *FDR* false discovery rate, *HG* histologic grade, *p.adjust* Benjamini and Hochberg FDR corrected *p* value of the overrepresentation test.

In the Clinseq data set, 8200 genes and 13,809 isoforms were found to be DE (FDR < 0.05) between HG1 and HG3 tumours, while there were few DE genes detected between HG1 and HG2 patients (Fig. 3a, b). If tumour size and lymph node status were adjusted, there were 7928 genes and 13,059 DE isoforms. In 3919 DE genes, the average expression level in HG3 was higher than that for HG1. In contrast, the average expression level in HG1 was higher than for HG3 in the other 4009 genes.

The numbers of DE genes between the HG2-High and HG2-Low groups for the five models (GGI, TG-Gene, TG-Iso, SC-Gene, and SC-Iso) were found to be 4091, 3750, 2935, 3750 and 3821, respectively. Between these sets, there were 1864 genes in common. Comparing the DE genes between HG2-High and HG2-Low to the 8200 DE genes between HG1 versus HG3, there were 2893, 2728, 2274, 2945 and 3001 genes in common. The large number of DE genes indicate that HG2-High and HG2-Low stratified by the TG models are biologically distinct.

The 13,809 DE isoforms between HG1 and HG3 were mapped to 9020 unique genes. Of these, 6052 already showed DE at the gene level (and were included in the 8200 DE genes). Hence, 2968 isoforms (of 2215 genes) were found to be DE and were not identified in the gene-level analysis (Fig. 3c). Pathway analysis revealed that most DE genes between HG1 and HG3 were enriched in cell-cycle pathways (Fig. 3d).

For 444 genes, DE isoforms of the same gene were associated with HG in opposite directions. This phenomenon was observed in 783 genes in the TCGA data set. Of these, 188 were common across the two data sets. For instance, there were six DE isoforms of gene *CD44* identified in both data sets. The average expression level in grade 1 tumours was lower than for grade 3 tumours in one isoform [Ensemble: ENST00000279452]. However, the average expression level of the other five isoforms was higher in HG1 than HG3. The expression levels of the *CD44* isoforms are illustrated in Additional file 1: Figure S6. Transcripts for this gene are determined by a complex alternative splicing mechanism that results in many functionally distinct isoforms. An association with *CD44* variant isoforms in the progression of head and neck squamous cell carcinoma has been reported [39].

For the three subcomponents of HG—tubularity, nuclearity and mitotic count—the numbers of DE genes between 1 and 3 were 1613, 165 and 10,617, respectively (see Additional file 1: Figure S5). The top overrepresented pathways for each component are listed in Additional file 1: Table S1. We found that cell-cycle pathways were also enriched among genes that were DE in tubularity and mitotic count. DE genes associated with nuclearity were found to be associated with the neurone system. This unlikely association might be a reflection of the modest number of DE genes. Interestingly, the scores of the three subcomponents of grade did not contribute equally to HG (see previous section), and they were also associated with different molecular mechanisms.

### Frequently selected predictors in prediction model are DE between HGs

To investigate if a smaller biomarker panel for prediction of TG could be defined, we tested whether the most frequently selected predictors (genes) over CV rounds could be utilised in a model and provide equally good predictions as the full model. The Clinseq TG-Gene model was cross-validated for 100 CV rounds. In each CV round, a regularised (elastic-net) regression model was fitted. Like the lasso regression model [40], the elastic-net model shrinks some of the coefficients to exactly zero, effectively performing variable selection. Here we utilised the property of variable selection in this model, but as we were concerned with robustness of the variable selection, we relied on the subsampling of the data that occurred during CV and ranked variables by how frequently they were selected over the CV rounds. We found that 10,454 genes were selected at least once. Ten gene sets with genes that were selected ≥99 to ≥90 (out of 100) CV rounds were fitted in ridge-penalised logistic regression and regular logistic regression models using the Clinseq data set, with the TCGA data set as an external test set to evaluate prediction performance (Additional file 1: Figure S7). A biomarker panel based on the 34 most frequently selected genes (the grade 34 panel) was the smallest panel that also provided maximal prediction performance when predicting TCGA individuals (AUC = 0.963, 95 % CI = 0.943–0.983; see also Additional file 1: Figure S7).

**Fig. 4:**
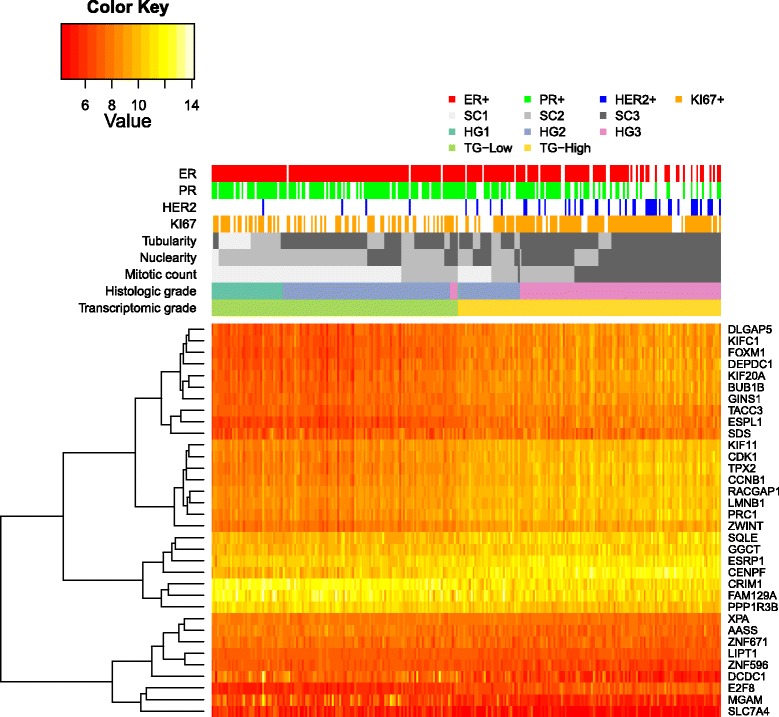
Heat map of 34 frequently selected genes in the TG-Gene model. In the Clinseq data set, the TG-Gene model was cross-validated 100 times to optimise parameters. In 100 rounds of CV, 34 genes were selected in the models in more than 92 out of the 100 CV rounds. Value of colour key is log2 (normalised RNA-seq count). *ER* oestrogen receptor, *PR* progesterone receptor, *RNA-seq* RNA sequencing

All of the 34 genes were DE between HG1 and HG3 (FDR adjusted *p*<0.05), and this gene set was mainly associated with cell-cycle-related pathways. The expression levels of the grade 34 panel plotted together with HG and TG also revealed visually distinct patterns of expression between TG groups (Fig. 4). The grade 34 panel provides a candidate set of genes that could be used to determine the TG in situations when transcriptome-wide data are not available. A list of the 34 genes is provided in Additional file 1: Table S2.

## Discussion

Sequencing-based cancer diagnostics may become routine in the clinic in the near future. This will enable more precise and accurate diagnosis of patients, and is likely to lead to a reduction of both over- and under-treatment of patients while also improving outcomes. Results from this study indicate that HG could be replaced by TG based on RNA-seq profiling. TG would also provide additional benefits through improved patient stratification by dichotomising the patients into low and high TG, and eliminating the intermediate group of HG2 patients. To our knowledge, this is the first comprehensive transcriptomic analysis of HG based on RNA-seq data where both gene- and isoform-level expression were considered.

Morphological and histologic classifications of breast cancer have been implemented in clinical settings for decades. The well-established association between disease progression and HGs [4] was confirmed in this study population. Our finding supports that HG is a prognostic indicator of breast cancer. In this study, we further provided molecular insight into HG. We confirmed previous findings that HG1 and HG3 tumours had distinct gene expression profiles [5]. Thousands of additional DE genes between HG1 and HG3 were identified compared to what was previously reported. We found the difference between HG1 and HG2 was ambiguous (Fig. 3a). This indicates that some HG2 patients have a similar expression profile as HG1 patients, suggesting that they may in fact be misclassified in the clinic, which could lead to over-treatment.

We demonstrated that multivariate prediction models using gene- or isoform-level RNA-seq data can be applied to discriminate between HG1 and HG3 tumours with high accuracy, and for further stratification of HG2 tumours into high and low TGs. Classification accuracy of the prediction models assessed by CV is high, with AUC = 0.975 (95 % CI = 0.968–0.983) in the SC-Iso model (Fig. 1a). The prediction model was also validated in a secondary data set (Fig. 1c) with equally good predictions (AUC = 0.970, 95 % CI = 0.951–0.990). Predictions for HG2 show a high degree of concordance across the five methods, while GGI has the most distinct profile compared to the other four prediction models. The differences in RFS rates between HG2-High and HG2-Low stratified by the TG-Gene model were statistically significant (Fig. 2, adjusted HR = 2.61, 95 % CI = 1.20–5.65). The distributions of PAM50 subtypes between TG and HG groups were found to be similar, providing further evidence that the TG model provides results that are concordant with HG (Fig. 2c and Additional file 1: Figure S3). Usually, luminal A is associated with lower grade and luminal B with higher grade [38]. The TG model classified HG2 patients so that the great majority of luminal A patients were labelled as TG-Low, while the great majority of luminal B cases were classified as TG-High. Moreover, the TG-Iso model was able to stratify luminal A patients into two groups with different recurrence rates (*p*=0.028, log-rank test). The proliferation indicator KI67 was also assessed in the two TG groups, and we found that it was higher in the predicted HG2-High group compared with the HG2-Low group. Our results suggest that our models are robust and consistent for prediction of TG and highly concordant in classification of HG1 and HG3.

In this study, we validated the GGI method proposed by Sotiriou et al. [5] for prediction of HG. We found that the GGI 97-gene signature had accuracy similar to the RNA-seq-based models developed in this study. However, GGI was indeed developed based on microarray technology and based on a relatively small amount of study material consisting of only ER-positive samples. The limitation of both the microarray technology and sample size was also reflected in the modest number of DE genes (97) detected in that study. In contrast, based on our RNA-seq data, we found 8200 DE genes. Applying the GGI algorithm to all of the 8200 DE genes would most likely introduce noise into the model and degrade the prediction accuracy of GGI. In developing the TG model, we applied a statistical learning approach based on regularised regression to select the most predictive genes, a strategy that is expected to outperform variable selection by filtering on *p* values from DE analysis. Moreover, RNA-seq data are expected to be less noisy compared to microarray-based expression profiling [41, 42], and therefore, they also have the potential to provide improved diagnostic models and biomarker panels. In this study, we found that the various methods (GGI versus TG) performed highly similarly, which is a positive result for those interested in translational applications as it indicates that grade can be predicted based on data from different technologies (microarray or RNA-seq) and using different models (GGI or TG) with high concordance.

We also proposed a biomarker panel consisting of 34 genes for prediction of TG. Compared with other signatures developed to predict grade, 15 of these 34 genes are common with the 96 genes used in the calculation of the GGI score [5]; none of them overlap with Ivshina’s five genes [43]. A recently published paper [44] using TCGA breast cancer RNA-seq data from 111 patients, developed a nine-gene panel to differentiate HG1 and HG2, and a 19-gene panel to classify HG2 and HG3. There is one gene from the grade 34 panel in the set of nine genes, and another one in the set of 19 genes. However, in this case it is not surprising that there are few common genes with the grade 34 panel since the models serve different purposes. Our TG model and the 34-gene set were developed for further stratifying HG2 tumours into poorly differentiated and well-differentiated tumours.

RNA-seq also enables isoform-level expression, while microarray quantification generally does not. In this study, we found 2115 DE isoforms between HG1 and HG3 that cannot be detected at the gene level. We also found that in 444 genes, isoforms of the same gene were associated with HG in opposite directions. This phenomenon was also observed in the TCGA data set. This indicates that isoforms of the same gene might be involved in different pathways conducting different functions. However, prediction models based on isoform-level data did not provide improved classification accuracy, although we note that classification between HG1 and HG3 groups is close to being perfect based on gene-level data.

Our study also provided some insights into subcomponents of HGs. There were significant differences between the three components. There were 10,617 genes DE between mitotic count scores 1 and 3. In contrast, there were only 165 DE genes identified between nuclear pleomorphism scores 1 and 3. The three components also contributed differently to the final prediction model. The AUC of mitotic count is higher than tubularity or nuclearity (0.92 versus 0.68 or 0.76), indicating that in the conventional histologic grading system, the mitotic count score has a stronger molecular signature at the RNA expression level compared with the tubularity and nuclearity scores.

## Conclusions

HG is an important indicator in routine breast cancer diagnostics. However, it is imperfect for patient stratification, particularly for patients with HG2 tumours. Here we demonstrated that RNA-seq expression profiling at gene and isoform level can be used to stratify HG2 tumours into two distinct groups with different prognostic outcomes, which has the potential to reduce both under- and over-treatment of breast cancer patients.

## Additional files

Additional file 1 All additional figures and tables listed. (PDF 488 kb)

Additional file 2 Includes lists of genes and coefficients of each model. (XLSX 1361 kb)

## Abbreviations

AUC: area under the ROC curve
CI: confidence interval
Clinseq: Clinical Sequencing of Cancer in Sweden
CV: cross-validation
DE: differential expression or differentially expressed
ER: oestrogen receptor
FDR, false discovery rate; FISH: fluorescent in situ hybridisation
GGI: genomic grade index
HER2: human epidermal growth factor receptor 2
HG: histologic grade
HR, hazard ratio; PR: progesterone receptor
RFS: recurrence-free survival
RNA-seq: RNA sequencing
ROC: receiver operating characteristic
TCGA: The Cancer Genome Atlas
TG: transcriptomic grade
