# Supplementary material for: Determining breast cancer histological grade from RNA-sequencing data
Source: Breast Cancer Res. 2016 May 10;18:48. doi: 10.1186/s13058-016-0710-8 (PMC4862203; doi:10.1186/s13058-016-0710-8)
Supplement: Additional file 1 — All additional figures and tables listed. (PDF 488 kb) [file 13058_2016_710_MOESM1_ESM.pdf]

Additional Figure 1

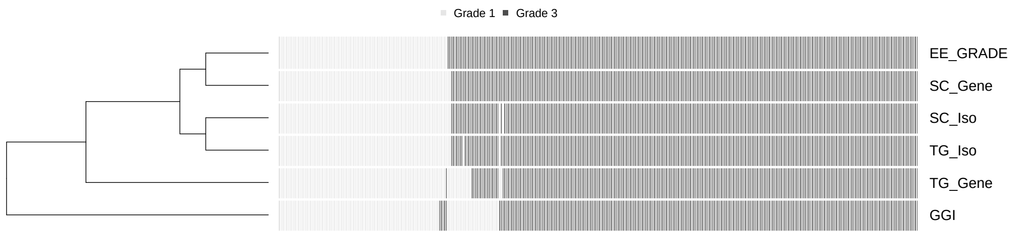

	GGI	TG_Gene	TG_Iso	SC_Gene	SC_Iso
Sensitivity	0.98	1.00	1.00	1.00	1.00
Specificity	0.76	0.86	0.96	0.98	0.96

Transcriptomic grades of HG1 and HG3 patients were predicted by five models (GGI, TG-Gene, TG-Iso, SC-Gene and SC-Iso), and compared with histologic grades. Results indicated a high degree of concordance across all methods, but with GGI being most different to the other models. Since the predictions were made by the model built in the same sample, whether statistical learning methods outperform GGI cannot be concluded due to the potential overfitting problem.

Additional Figure 2

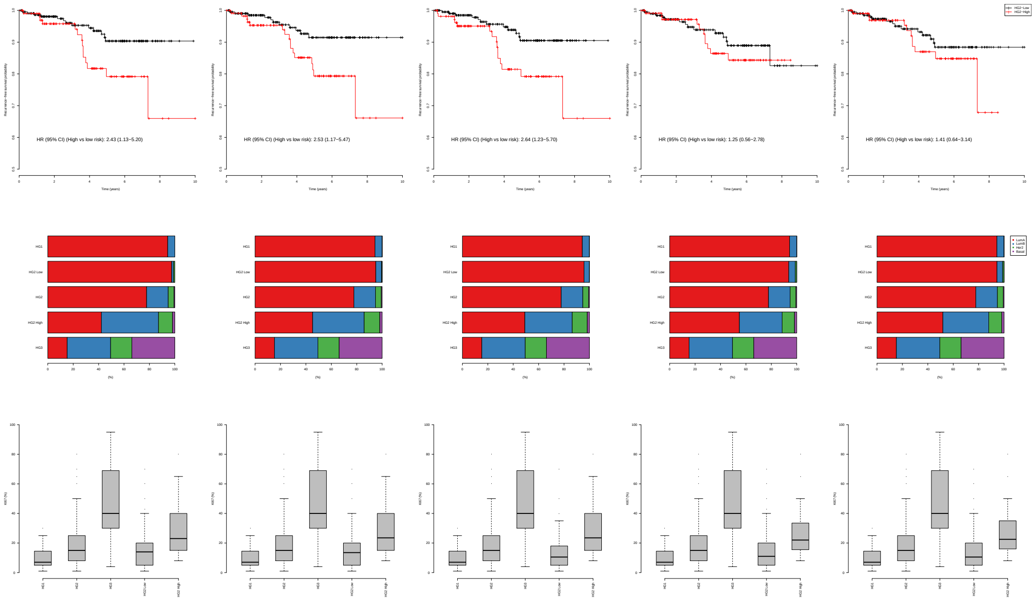

Figures in the first row are recurrence-free Kaplan-Meir curves of HG2-High and HG2-Low groups by five models (GGI, TG-Gene, TG-Iso, SC-Gene, SC-Iso) in patients with histologic grade 2 tumours. Figures in the second row are PAM50 subtypes distribution by five models. Figures in the third row are KI67 distribution by five models. “HG2 Low” and “HG2 High” groups are predicted by five models in patients with histologic grade 2 tumours. Five models from left to right: GGI model, TG-Gene model, TG-Iso model, SC-Gene model and SC-Iso model. Sample from Clinseq and TCGA dataset were combined.

Additional Figure 3

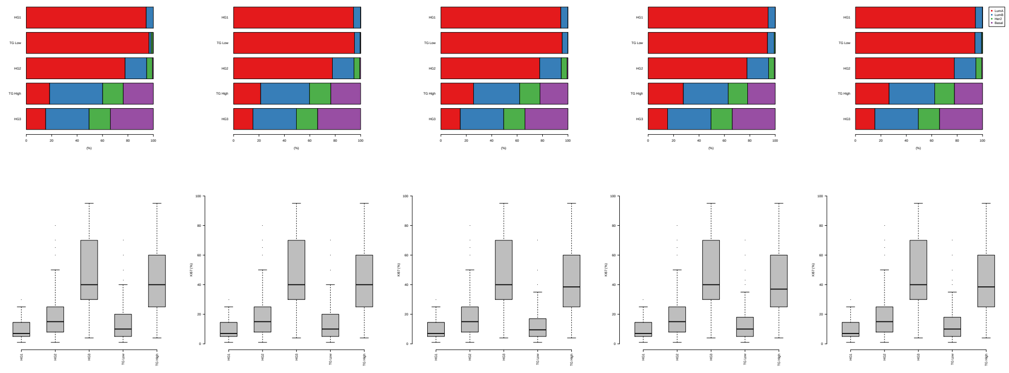

Figures in the first row are PAM50 subtypes distribution by five models (GGI, TG-Gene, TG-Iso, SC-Gene, SC-Iso). TG-High and TG-Low groups were predicted by five models in all the patients from both Clinseq and TCGA datasets. Figures in the second row are KI67 distribution by five models in all the samples from both Clinseq and TCGA datasets. Five models from left to right: GGI model, TG-Gene model, TG-Iso model, SC-Gene model and SC-Iso model. Sample from Clinseq and TCGA dataset were combined.

Additional Figure 4

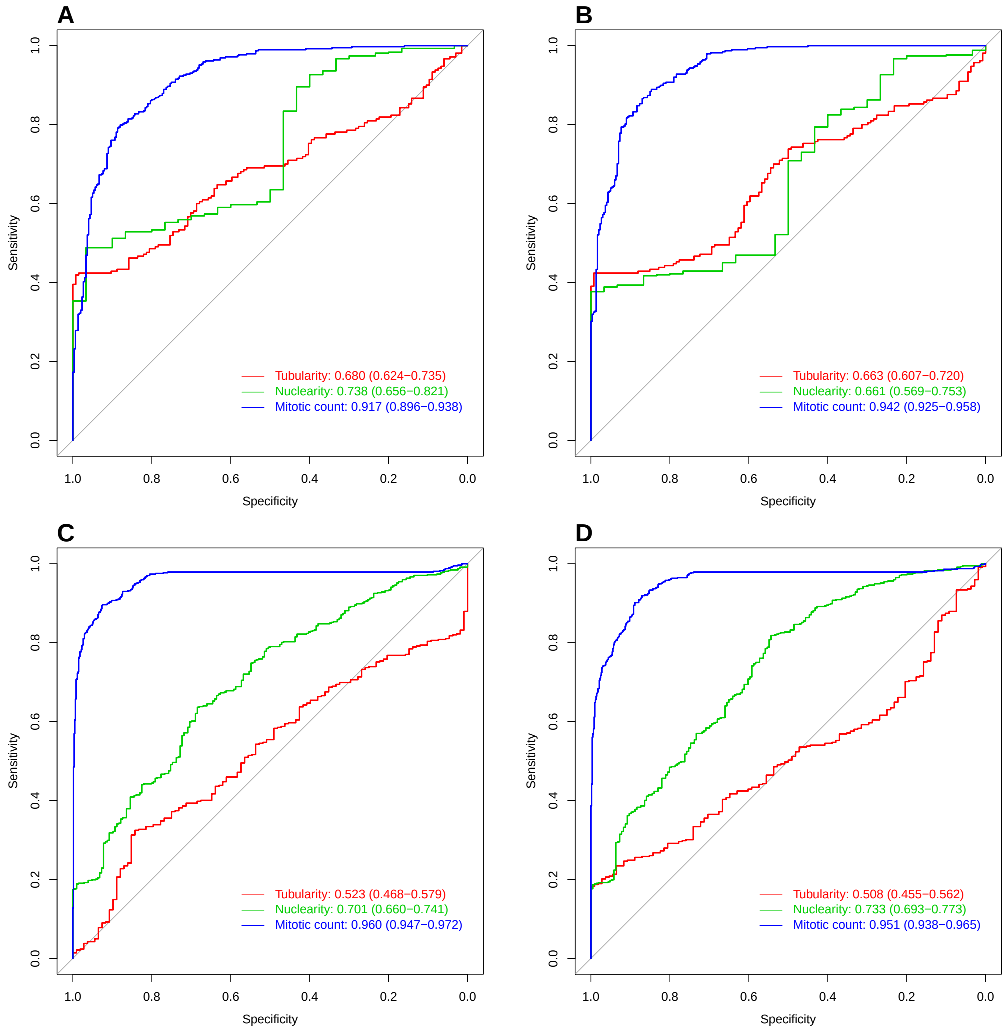

ROC curves of three subcomponents. (A) SC-Gene model in Clinseq dataset; (B) SC-Gene model in TCGA dataset; (C) SC-Iso model in Clinseq dataset; (D) SC-Iso model in TCGA dataset. AUC of ROC curves and 95% CI were listed in each plot.

Additional Figure 5

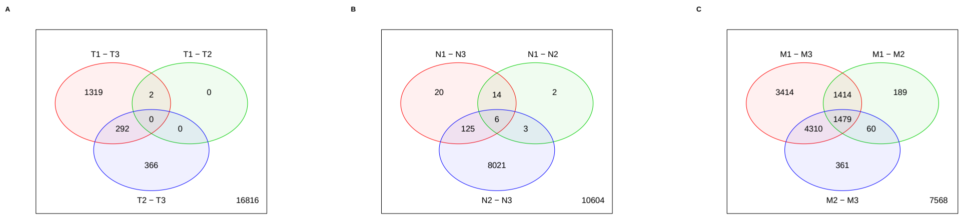

Venn diagram of DE genes for three subcomponents of histologic grade. (A) Tubularity; (B) nuclearity; (C) mitotic counts. Each subcomponent was scored from 1 to 3 according to Nottingham criteria. In each subcomponent, differential expression was analysed among sub-scores.

Additional Figure 6

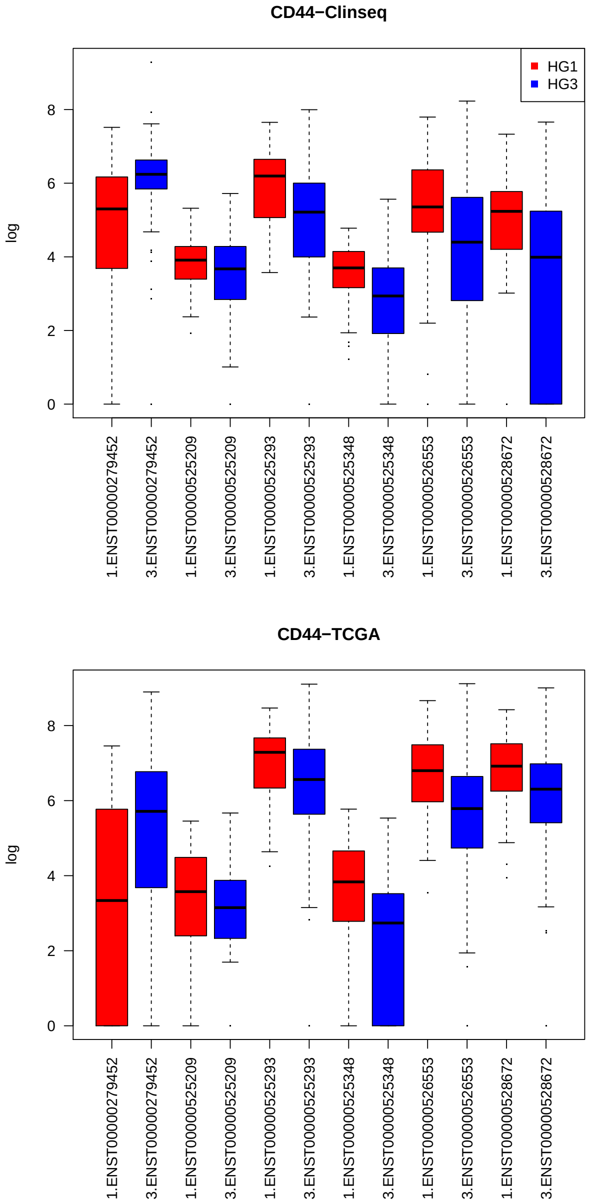

Expression level of CD44 isoforms in Clinseq and TCGA dataset. There were six DE isoforms of gene *CD44* identified in both datasets. The average

expression level in grade 1 tumours was lower than grade 3's in one isoforms (Ensemble transcript ID: ENST00000279452). However, the average expression level of the other five isoforms was higher in HG1 than HG3's.

Additional Figure 7

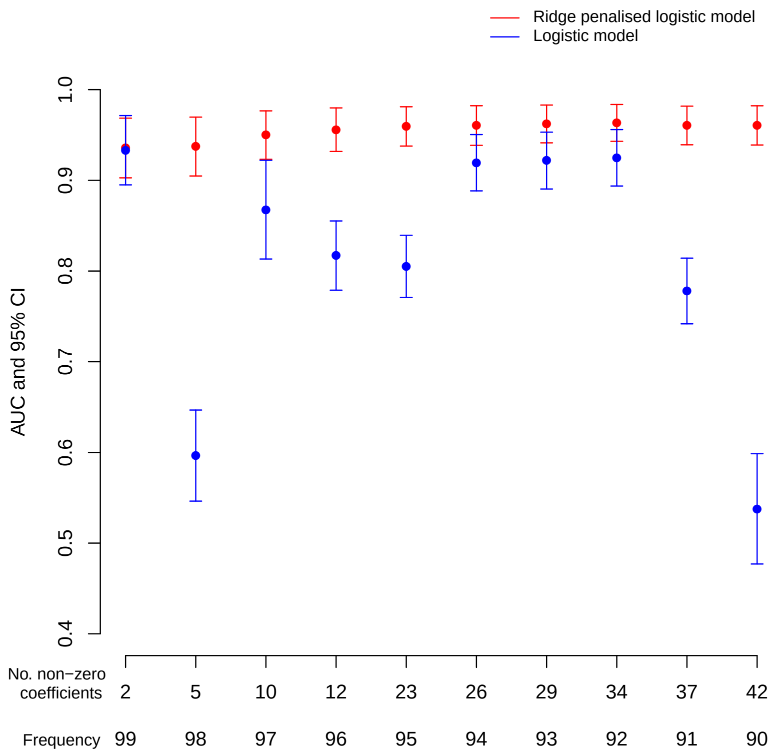

We tested whether the most frequently been selected genes could be utilised as a minimal gene panel. 10 gene sets from genes been selected $\geq$ 99 to $\geq$ 90 rounds of CV were fitted Ridge-penalised logistic regression and regular logistic regression models in Clinseq. Predictions in TCGA dataset were made for each model. AUC and 95% CI of each gene set were plotted in Additional Figure 5. For regular logistic regression model, predictions of models with less than 26 predictors were unstable. Model accuracy dropped when noise introduced by more than 34 predictors. For Ridge-penalised logistic regression model, the highest AUC was achieved when model has 34 genes.

Additional Figure 8

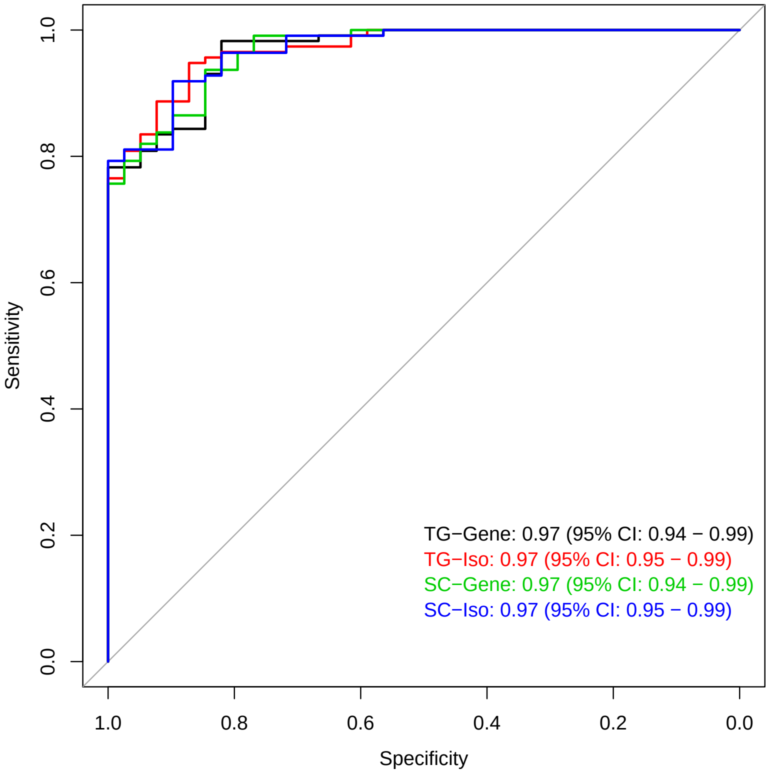

Cross-dataset validation of multivariate prediction models (TG-Gene, TG-Iso, SC-Gene and SC-Iso). Models were estimated based on the TCGA dataset, and grade in the Clinseq dataset was predicted.

Additional Figure 9

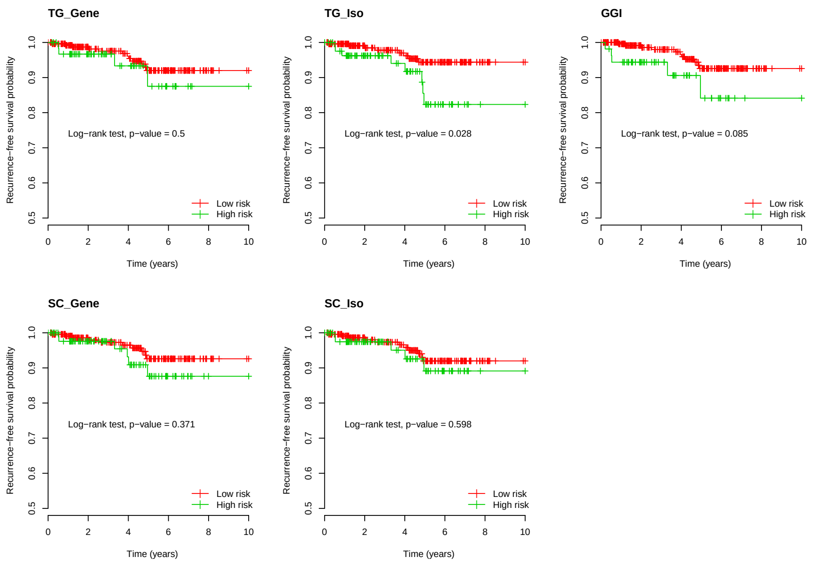

Kaplan-Meir curves of RFS between High and Low risk groups stratified by models (TG-Gene, TG-Iso, GGI, SC-Gene and SC-Iso) within subtype luminal A.

Additional Figure 10

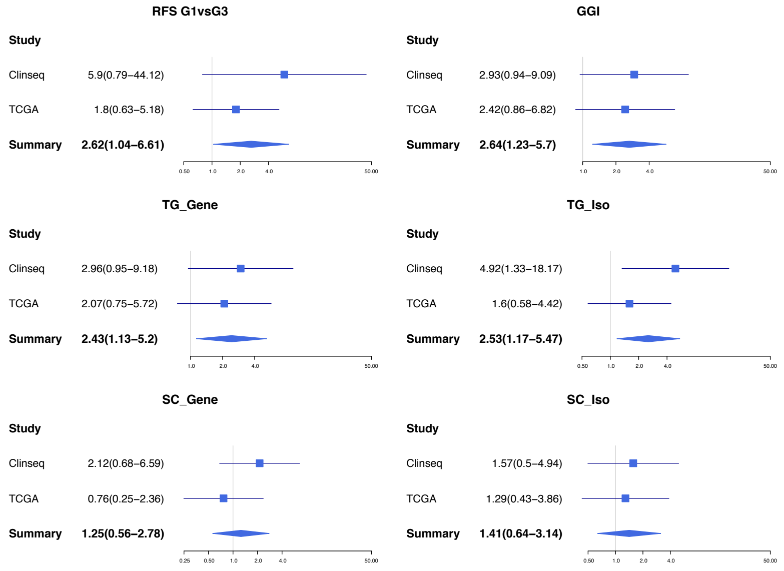

Forest plots of univariate cox-regression model comparing Grade 1 and 3 or predicted High and Low risk group of models (GGI, TG-Gene, TG-Iso, SC-Gene and SC-Iso). The summarised HR was cox regression estimation stratified by dataset, thus allowing for different baseline hazard functions between cohorts.

Additional Figure 11

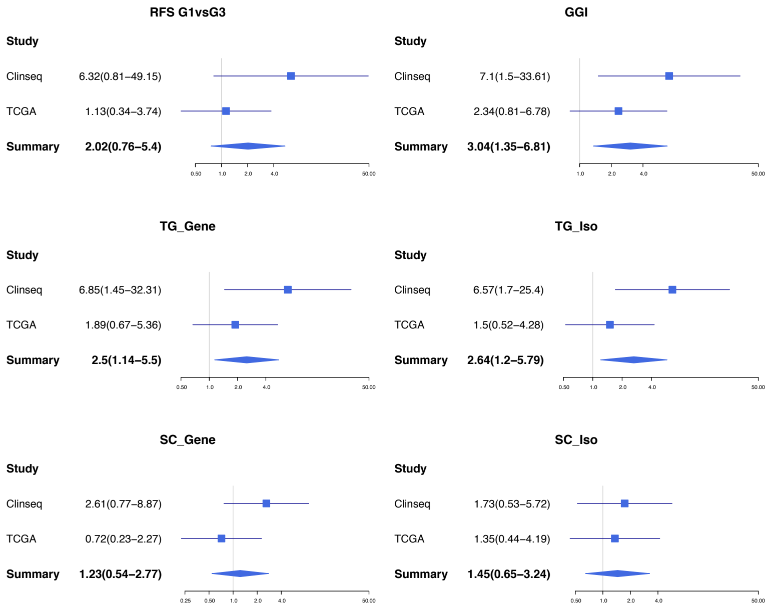

Forest plots of multi-variate cox-regression model comparing Grade 1 and 3 or predicted High and Low risk group of models (GGI, TG-Gene, TG-Iso, SC-Gene and SC-Iso), adjusted for age, tumour size, lymph node status and ER status. The summarised HR was cox regression estimation stratified by dataset, thus allowing for different baseline hazard functions between cohorts.

Additional Table 1

The top pathways of DE genes in three subcomponents of histologic grade

Reactome ID	Pathway	GeneRatio	BgRatio	pvalue	p.adjust
**Tubularity**					
1640170	Cell Cycle	182/668	554/6958	3.67e-58	1.25e-55
69278	Cell Cycle, Mitotic	169/668	489/6958	2.93e-57	5.00e-55
453277	Mitotic M-M/G1 phases	123/668	346/6958	2.61e-42	2.97e-40
68886	M Phase	102/668	314/6958	4.68e-31	4.00e-29
68877	Mitotic Prometaphase	60/668	125/6958	5.37e-29	3.67e-27
2500257	Resolution of Sister Chromatid Cohesion	54/668	116/6958	2.39e-25	1.36e-23
2555396	Mitotic Metaphase and Anaphase	70/668	189/6958	3.21e-25	1.57e-23
68882	Mitotic Anaphase	69/668	188/6958	1.35e-24	5.77e-23
2467813	Separation of Sister Chromatids	64/668	177/6958	1.88e-22	7.13e-21
453279	Mitotic G1-G1/S phases	52/668	133/6958	3.50e-20	1.20e-18
**Nuclearity**					
112315	Transmission across Chemical Synapses	6/59	196/6958	6.03e-03	6.03e-02
112316	Neuronal System	6/59	275/6958	2.82e-02	1.41e-01
**Mitotic counts**					
69242	S Phase	96/3899	118/6958	4.51e-09	2.49e-06
453279	Mitotic G1-G1/S phases	106/3899	133/6958	6.52e-09	2.49e-06
69239	Synthesis of DNA	78/3899	93/6958	8.94e-09	2.49e-06
1236975	Antigen processing-Cross presentation	65/3899	75/6958	1.06e-08	2.49e-06
69306	DNA Replication	82/3899	99/6958	1.22e-08	2.49e-06
69206	G1/S Transition	87/3899	107/6958	2.56e-08	4.35e-06
69278	Cell Cycle, Mitotic	328/3899	489/6958	1.57e-07	2.29e-05
1640170	Cell Cycle	367/3899	554/6958	2.15e-07	2.74e-05
68874	M/G1 Transition	64/3899	77/6958	3.93e-07	4.01e-05
69002	DNA Replication Pre-Initiation	64/3899	77/6958	3.93e-07	4.01e-05

Additional Table 2

34 gene list

ensembl_gene_id	hgnc_symbol	chromosome_name	start_position	end_position	band	strand
ENSG00000083814	ZNF671	19	57719751	57727624	q13.43	-1
ENSG00000198901	PRC1	15	90966038	90995629	q26.1	-1
ENSG00000170312	CDK1	10	60778331	60794852	q21.2	1
ENSG00000122952	ZWINT	10	56357228	56361275	q21.1	-1
ENSG00000113368	LMNB1	5	126776623	126837020	q23.2	1
ENSG00000173281	PPP1R3B	8	9136255	9151574	p23.1	-1
ENSG00000088325	TPX2	20	31739271	31801805	q11.21	1
ENSG00000111206	FOXM1	12	2857681	2877040	p13.33	-1
ENSG00000161800	RACGAP1	12	49976923	50033136	q13.12	-1
ENSG00000104549	SQLE	8	124998497	125022283	q24.13	1
ENSG00000144182	LIPT1	2	99154955	99163157	q11.2	1
ENSG00000117724	CENPF	1	214603195	214664588	q41	1
ENSG00000138160	KIF11	10	92593286	92655395	q23.33	1
ENSG00000104413	ESRP1	8	94641074	94707466	q22.1	1
ENSG00000156970	BUB1B	15	40161023	40221136	q15.1	1
ENSG00000136936	XPA	9	97674909	97697357	q22.33	-1
ENSG00000150938	CRIM1	2	36355926	36551135	p22.2	1
ENSG00000134057	CCNB1	5	69167010	69178245	q13.2	1
ENSG00000170959	DCDC1	11	30830369	31369810	p13	-1
ENSG00000237649	KIFC1	6	33391536	33409924	p21.32	1
ENSG00000099960	SLC7A4	22	21028718	21032840	q11.21	-1
ENSG00000013810	TACC3	4	1721490	1745176	p16.3	1
ENSG00000129173	E2F8	11	19224063	19241620	p15.1	-1
ENSG00000008311	AASS	7	122075647	122144280	q31.32	-1
ENSG00000112984	KIF20A	5	138178719	138187715	q31.2	1
ENSG00000006625	GGCT	7	30496621	30551479	p14.3	-1
ENSG00000135094	SDS	12	113392445	113426301	q24.13	-1
ENSG00000257335	MGAM	7	141907813	142106747	q34	1
ENSG00000135842	FAM129A	1	184790724	184974550	q25.3	-1
ENSG00000101003	GINS1	20	25407727	25452628	p11.21	1
ENSG00000172748	ZNF596	8	232137	247342	p23.3	1
ENSG00000126787	DLGAP5	14	55148112	55191678	q22.3	-1
ENSG00000024526	DEPDC1	1	68474152	68497221	p31.3	-1
ENSG00000135476	ESPL1	12	53268299	53293643	q13.13	1

Additional Table 3

P-value of Log-rank test and HRs of cox-regression on recurrence-free survival comparing breast cancer patients with different histologic grades and predicted groups in grade 2 tumours in Clinseq dataset

Clinseq	N	Events	Log-rank test p-value	HR unadjusted† (95% CI)	HR adjusted‡ (95% CI)
**Histologic grades**					
HG1	39	1	0.049*	1.00 (Reference)	1.00 (Reference)
HG3	115	19		5.90 (0.79-44.12)	6.32 (0.81-49.15)
**GGI**					
Low risk	90	6	0.051	1.00 (Reference)	1.00 (Reference)
High risk	31	6		2.93 (0.94-9.09)	7.10 (1.50-33.61)*
**TG-Gene**					
Low risk	89	6	0.049*	1.00 (Reference)	1.00 (Reference)
High risk	32	6		2.96 (0.95-9.18)	6.85 (1.45-32.31)*
**TG-Iso**					
Low risk	74	3	0.008*	1.00 (Reference)	1.00 (Reference)
High risk	47	9		4.92 (1.33-18.17)*	6.57 (1.70-25.40)*
**SC-Gene**					
Low risk	81	6	0.183	1.00 (Reference)	1.00 (Reference)
High risk	40	6		2.12 (0.68-6.59)	2.61 (0.77-8.87)
**SC-Iso**					
Low risk	82	7	0.440	1.00 (Reference)	1.00 (Reference)
High risk	39	5		1.57 (0.50-4.94)	1.73 (0.53-5.72)

† HR unadjusted;

‡ HR adjusted for age, tumour size, lymph node status and ER status;

$*$ p-value < 0.05;

Additional Table 4

P-value of Log-rank test and HRs of cox-regression on recurrence-free survival comparing breast cancer patients with different histologic grades and predicted groups in grade 2 tumours in TCGA dataset

TCGA	N	Events	Log-rank test p-value	HR unadjusted† (95% CI)	HR adjusted‡ (95% CI)
**Histologic grades**					
HG1	59	5	0.268	1.00 (Reference)	1.00 (Reference)
HG3	179	25		1.80 (0.63-5.18)	1.13 (0.34-3.74)
**GGI**					
Low risk	133	6	0.083	1.00 (Reference)	1.00 (Reference)
High risk	79	9		2.42 (0.86-6.82)	2.34 (0.81-6.78)
**TG-Gene**					
Low risk	139	7	0.150	1.00 (Reference)	1.00 (Reference)
High risk	73	8		2.07 (0.75-5.72)	1.89 (0.67-5.36)
**TG-Iso**					
Low risk	142	8	0.362	1.00 (Reference)	1.00 (Reference)
High risk	70	7		1.6 (0.58-4.42)	1.5 (0.52-4.28)
**SC-Gene**					
Low risk	117	8	0.640	1.00 (Reference)	1.00 (Reference)
High risk	77	5		0.76 (0.25-2.36)	0.72 (0.23-2.27)
**SC-Iso**					
Low risk	126	7	0.652	1.00 (Reference)	1.00 (Reference)
High risk	68	6		1.29 (0.43-3.86)	1.35 (0.44-4.19)

† HR unadjusted;

‡ HR adjusted for age, tumour size, lymph node status and ER status;

$*$ p-value < 0.05;
